# Long-term effects of smoking on tooth loss after cessation among middle-aged Finnish adults: the Northern Finland Birth Cohort 1966 Study

**DOI:** 10.1186/s12889-016-3556-1

**Published:** 2016-08-24

**Authors:** Toni Similä, Juha Auvinen, Markku Timonen, Jorma I. Virtanen

**Affiliations:** 1 Research Unit of Oral Health Sciences, Faculty of Medicine, University of Oulu, P.O. Box 5281, Oulu, FIN-90014 Finland; 2 Medical Research Center Oulu, Oulu University Hospital, Oulu, Finland; 3 Center for Life Course Health Research, University of Oulu, Oulu, Finland

**Keywords:** Smoking, Smoking cessation, Tobacco, Tooth loss, Adult

## Abstract

**Background:**

Despite smoking cessation efforts, cigarette smoking remains a serious general and oral health problem. We aimed to investigate the putative benefits of smoking cessation on dentition and to analyse whether the time elapsed since smoking cessation associated positively with the remaining number of teeth.

**Methods:**

This cross-sectional study analyses data from the 46-year follow-up of the Northern Finland Birth Cohort Study 1966 (NFBC1966). A total of 5 540 subjects participated in this cross-sectional study, which utilises both clinical dental examinations and mailed questionnaires. We used the following information on smoking: status (current, former, never), years of smoking (current, former) and years elapsed since smoking cessation (former). Self-reported and clinically measured number of teeth (including third molars) served as alternative outcomes. We used binary logistic regression models to analyse the dichotomised number of teeth (‘0–27’, ’28–32’) and then calculated unadjusted and adjusted odds ratios (OR) with 95 % confidence intervals (CI) for the smoking variables (never smoker as the reference). Gender, education, tooth brushing frequency, diabetes and alcohol use served as confounders for the adjusted models.

**Results:**

Ten years or more of smoking associated with tooth loss; this effect was the strongest among men who reported having an ongoing smoking habit (self-reported outcome: adjusted OR = 1.74, CI = 1.40–2.16) and the weakest among women classified as former smokers (self-reported outcome: adjusted OR = 1.27, CI = 1.00–1.62).

**Conclusions:**

This study shows that smoking has long-term effects on tooth loss even after cessation. The findings support smoking cessation efforts to reduce oral health risks.

## Background

Cigarette smoking is a major public health problem worldwide. Various efforts, such as legislative activities, public health programmes and drug therapies promote smoking cessation [1]. Yet, despite these efforts, cigarette smoking remains a serious general and oral health problem, as smoking associates not only with health, but also with overall quality of life [2, 3].

Smoking is a confirmed risk factor for a number of oral diseases and conditions, such as periodontitis and oral cancer, and associates with dental caries, tooth loss and implant failure [4–7]. Smoking associates negatively with the number of teeth such that a person with a high burden of smoking history is more likely to have fewer remaining teeth [8–10].

The health benefits of smoking cessation are well known, and quitting even in middle-age can lower the overall disease risk [11]. Consequently, smoking cessation has many benefits for oral health, but a long period of abstinence from smoking is necessary to approach a similar risk level for tooth loss as that of never smokers [12–15]. Nevertheless, detailed studies regarding the oral health of former smokers among the Western adult population are rare.

A recent study of middle-aged Finns found that pack-years and years smoked associated with the number of missing teeth in an exposure-dependent manner: 11–20 pack-years and 21–30 years of smoking significantly raised the probability of tooth loss over never smokers, and the effect was even stronger among those with higher pack-years and a longer duration of smoking [8]. However, that study investigated the matter no further among former smokers.

Among middle-aged Finns with good oral health and access to subsidised dental care since childhood [16], our primary aim was to investigate the putative benefits of smoking cessation on dentition and, secondly, whether the time elapsed since smoking cessation positively associates with the remaining number of teeth.

## Methods

### Study design

This cross-sectional study used data from the longitudinal Northern Finland Birth Cohort Study 1966 (NFBC1966), which consists of a comprehensive sample of individuals from the two northernmost provinces of Finland (Lapland and Oulu) whose expected year of birth was 1966 (12 068 mothers, 12 231 children, 96.3 % of all births in this region) [17]. The cohort members have attended regular monitoring since their mothers were pregnant. The Ethics Committee of the Northern Ostrobothnia Hospital District in Oulu, Finland approved the study protocol, which followed the principles of the Declaration of Helsinki. The participants’ participation was voluntary and all provided their written informed consent. The data were handled on a group level only, and identification codes replaced the personal information.

This study used data from the 46-year follow-up (carried out in 2012–2014), which included a mailed survey and a comprehensive in-person clinical health examination. The questionnaires and invitations to health examinations were mailed to all who lived in Finland and whose addresses were known at the beginning of 2012 (*n* = 10 321). Subjects living in municipalities within 100 km of the city of Oulu (Oulu subpopulation, *n* = 3135) were invited to more intensive health examinations, which also included a full inspection of the mouth and teeth. In all, 5 540 participants (participation rate 54 %) provided full information on smoking and self-reported the number of teeth in mailed questionnaire. The clinical dental examination for the Oulu subpopulation provided information on the dentition of 1 891 participants (participation rate 60 %).

### Smoking

The mailed questionnaire used the following questions to enquire about previous and current smoking habits at the age of 46: “Have you ever smoked in your life? (Yes, I started at the age of XX; No)”, “Have you ever smoked regularly? (Yes, I have smoked regularly for XX years; No)”, “If you quit smoking, how old you were when you did?” and “Do you currently smoke? (Not at all – Seven days a week)”. We used separate categories for current, former and never smokers. Here, ‘current smokers’ were those who reported smoking at least occasionally. ‘Former smokers’ included those who had smoked daily for at least one year, but had quit smoking and were not smokers at the time of the study. ‘Never smokers’ included all participants who had smoked daily for less than one year in their lifetime and were not smokers at the time of the follow-up.

Years of smoking served as a measure of smoking history among current and former smokers. We also measured years elapsed since smoking cessation among former smokers.

We did not distinguish between the reported use of different tobacco products (of the entire study sample, 150 participants reported smoking other tobacco products than filtered cigarettes) in our analyses.

### Number of teeth

Subjects reported the number of teeth they had at the age of 46 with no distinction for third molars from other teeth. We used this measure as the main outcome and the dichotomised number of teeth (‘0–27’ or ‘28–32’) for the binary logistic regression analyses. We validated self-reported outcome with corresponding information on the number of teeth from the clinical examinations (*n* = 1 674). Our previous paper includes details on this clinical measure [8].

### Confounders

According to previous research, we defined education, tooth brushing frequency, alcohol use, and diabetes as potential confounders [8, 10, 12].

Subjects reported in the mailed questionnaire their level of education at the age of 46; we then used this information to form a three-class ordinal variable: 1) ‘basic education’ included those who had not graduated from high school and had no formal vocational qualification, 2) ‘secondary education’ included those who had graduated from high school or vocational school, and 3) ‘higher education’ included participants with a university degree or who had graduated from a polytechnic or equivalent school.

The questionnaire included the frequency of tooth brushing, for which we formed two categories based on the general recommendation to brush twice daily: ‘once daily or less’ or ‘at least twice daily’ [18]. The questionnaire enquired about alcohol use with several separate questions on the frequencies and the number of standard doses of different beverages (beer, cider and long drink; wine and spirits) consumed. We used Sundell et al.’s [19] classification for alcohol content per standard dose of different beverages and calculated a continuous grammes-per-week variable. We defined moderate to heavy alcohol users as those men consuming > 230 g/week and women consuming > 150 g/week; the rest were defined as light drinkers or non-users.

We defined doctor-diagnosed diabetes by combining and verifying information from numerous sources: participants’ self-reported type 1 and 2 diabetes diagnoses and medications, hospital outpatient and inpatient registers, and medication registers from the Social Insurance Institution of Finland. These registers include diagnoses made by doctors. We used a dichotomous variable (yes/no) and did not distinguish type 1 from type 2 diabetes.

### Statistical analysis

We used a binary logistic regression model to analyse the dichotomised number of teeth (‘0–27’, ’28–32’) and then calculated unadjusted and adjusted odds ratios (OR) with 95 % confidence intervals (CI) for each smoking category. We also performed stratified analyses by gender.

For validity analyses of self-reported number of teeth, we calculated Cohen’s kappa values for agreement between dichotomised versions of the clinically assessed number of teeth (as the true condition) and the self-reported number of teeth by the study variables.

We used the statistical package R environment version 3.1.2 for all statistical analyses [20]. For the binary logistic regression modelling, we used the glm function with default options. For Cohen’s kappa calculations, we used the cohen.kappa function in the psych package.

## Results

Table 1 shows the distribution, by smoking status, of the participants who participated in the questionnaire survey. Almost half of them were never smokers and the majority were women (60 %), whereas among current smokers and former smokers, men and women were more evenly distributed (52 % of both current smokers and former smokers were men). Education, tooth brushing frequency, and alcohol use appeared to associate with smoking status. The percentages of those with higher education, who brushed their teeth at least twice daily, and who consumed alcohol lightly or not at all were the highest among never smokers, but lower among former smokers and the lowest among current smokers. The number of teeth varied with smoking status: the percentages of those with fewer than 28 teeth among current smokers, former smokers, and never smokers were 49 %, 42 % and 35 %, respectively.

**Table 1:** Distribution of the participants’ smoking status and other study variables (*n* = 6344)

	Never smoker* (*n* = 3062)		Former smoker** (*n* = 1525)		Current smoker*** (*n* = 1757)	
	%	*n*	%	*n*	%	*n*
Gender (*n* = 6344)						
Male	40	1213	52	786	52	916
Female	60	1849	48	739	48	841
Education (*n* = 6248)						
Basic	4	114	8	112	11	197
Secondary	27	817	40	603	46	794
Higher	69	2094	52	783	43	734
Tooth brushing (*n* = 6324)						
Once daily or less	28	862	38	573	45	782
At least twice daily	72	2191	62	947	55	969
Alcohol use, g/week (*n* = 6336)						
Moderate to heavy drinker^a^	5	162	11	165	19	327
Non-drinker or light drinker^b^	95	2895	89	1359	81	1428
Diabetes (*n* = 6256)						
Yes	3	103	4	65	5	85
No	97	2925	96	1423	95	1655
Number of teeth^c^ (*n* = 5540)						
0–27	35	933	42	559	49	742
28–32	65	1763	58	785	51	758

*Those who had never smoked, or have smoked less than a year and did not smoke at the time of the survey

**Those who have smoked at least for a year but did not smoke at the time of the survey

***Those who smoked at the time of the survey

^a^>230 g/week for men and >150 g/week for women

^b^0–230 g/week for men and 0–150 g/week for women

^c^Including third molars

Table 2 displays self-reported information on explanatory variables and the number of teeth by gender among the former smokers. More men than women had a long smoking history, whereas women had a longer time since smoking cessation. Totally 58 % of the former smokers had at least 28 teeth in their mouth. The mean number of teeth among men was 27.0 compared to 27.4 among women. Years since smoking cessation and years of smoking appeared to associate with the number of teeth among men, but not among women.

**Table 2:** Self-reported number of teeth by gender and other study variables among former smokers

	Male (*n* = 670)			Female (*n* = 674)			Total (*n* = 1344)		
	Participants	Number of teeth		Participants	Number of teeth		Participants	Number of teeth	
	%	Mean	≥28: %	%	Mean	≥28: %	%	Mean	≥28: %
Years since smoking cessation (*n* = 1344)									
0–9 years	44	26.4	51	37	27.3	62	40	26.8	56
10–19 years	31	27.4	61	28	27.5	62	30	27.4	61
20 or more years	25	27.7	62	35	27.4	56	30	27.5	58
Years of smoking (*n* = 1324)									
1–9 years	28	27.6	65	46	27.5	62	37	27.6	63
10–19 years	34	27.1	57	29	27.5	56	32	27.3	57
20 or more years	38	26.4	51	25	27.1	61	31	26.7	56
Education (*n* = 1319)									
Basic	9	26.8	47	5	25.9	33	7	26.4	42
Secondary	47	26.4	51	31	27.0	52	39	26.7	52
Higher	44	27.8	67	64	27.7	65	54	27.7	65
Tooth brushing (*n* = 1340)									
Once daily or less	49	26.3	49	24	27.1	57	36	26.6	52
At least twice daily	51	27.7	65	76	27.5	61	64	27.6	62
Alcohol use, g/week (*n* = 1343)									
Moderate to heavy drinker	16	27.4	68	6	27.7	70	11	27.5	68
Non-drinker or light drinker	84	27.0	55	94	27.4	59	89	27.2	57
Diabetes (*n* = 1312)									
Yes	4	27.4	54	4	27.2	58	4	27.3	56
No	96	27.1	58	96	27.4	60	96	27.2	59
Total (*n* = 1344)	100	27.0	57	100	27.4	60	100	27.2	58

Table 3 shows the validity calculations for self-reported number of teeth and the clinically verified findings of the sub-sample. The estimated agreement for the sample according to Cohen’s kappa analyses was 0.78 with minor variation among the categories of the study variables (0.74–0.85); for instance, the estimated agreement among women was 0.79 and among men 0.76.

**Table 3:** Agreement between self-reported and clinically assessed number of teeth (‘0–27’, ‘28–32’) by study variables

	*n*	Cohen’s kappa	95 % CI
Gender			
Male	745	0.76	0.72–0.81
Female	929	0.79	0.75–0.83
Education			
Basic	79	0.85	0.73–0.96
Secondary	584	0.74	0.69–0.79
Higher	988	0.79	0.75–0.83
Tooth brushing			
Once daily or less	549	0.80	0.75–0.85
At least twice daily	1123	0.77	0.73–0.81
Smoking status			
Never	784	0.78	0.73–0.82
Former	400	0.82	0.76–0.88
Current	360	0.75	0.68–0.82
Alcohol use, g/week			
Moderate to heavy drinker	170	0.74	0.64–0.85
Non-drinker or light drinker	1504	0.78	0.75–0.81
Diabetes			
Yes	67	0.82	0.68–0.96
No	1586	0.78	0.75–0.81
Total	1674	0.78	0.75–0.81

Results are presented with Cohen’s kappa values and their 95 % confidence intervals

Table 4 shows the results of the binary logistic regression analyses of the length of smoking history and the self-reported number of teeth. Ten or more years of smoking associated with fewer teeth (fewer than 28 teeth) than among never smokers irrespective of current smoking status and gender. This effect was the strongest among men who reported having an ongoing smoking habit (adjusted OR = 1.74, CI = 1.40–2.16), though a weak effect was evident even among former-smoker women (adjusted OR = 1.27, CI = 1.00–1.62). Corresponding analyses based on the clinically assessed number of teeth yielded similar associations.

**Table 4:** Results of the binary logistic regression analyses, with self-reported number of teeth as the outcome (*n* = 5245)

Smoking status	Gender	Years of smoking (ref. = Never smoker)	Unadjusted		Adjusted*	
			OR	95 % CI	OR	95 % CI
Current	Male					
		1–9 (*n* = 74)	1.43	0.88–2.30	1.38	0.85–2.25
		10+ (*n* = 621)	2.17	1.76–2.66	1.74	1.40–2.16
	Female					
		1–9 (*n* = 101)	1.20	0.80–1.82	1.13	0.74–1.72
		10+ (*n* = 559)	1.80	1.48–2.18	1.65	1.34–2.02
	Total					
		1–9 (*n* = 175)	1.29	0.95–1.77	1.21	0.88–1.67
		10+ (*n* = 1180)	1.99	1.73–2.29	1.70	1.46–1.97
Former	Male					
		1–9 (*n* = 180)	0.98	0.70–1.37	0.93	0.66–1.30
		10+ (*n* = 458)	1.53	1.22–1.92	1.35	1.07–1.71
	Female					
		1–9 (*n* = 287)	1.15	0.89–1.49	1.12	0.86–1.46
		10+ (*n* = 338)	1.35	1.06–1.72	1.27	1.00–1.62
	Total					
		1–9 (*n* = 467)	1.09	0.88–1.33	1.04	0.85–1.28
		10+ (*n* = 796)	1.46	1.24–1.71	1.31	1.11–1.55

Unadjusted and adjusted odds ratios (OR) with 95 % confidence intervals (CI) (28–32 teeth as the reference)

*Adjusted for gender, education, tooth brushing, alcohol use (g/week) and diabetes

**Fig. 1:**
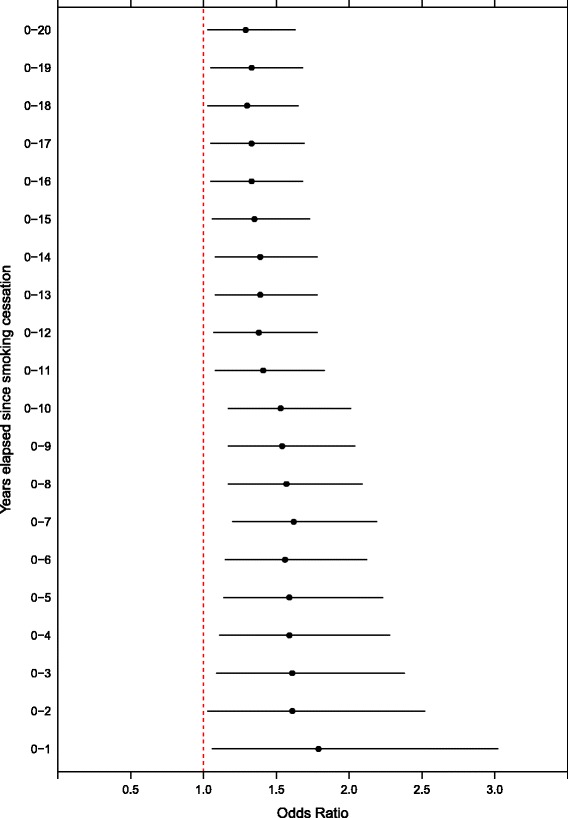
Association between smoking cessation and the number of teeth along with odds ratios among men. Fewer than 28 teeth served as the reference for the outcome. Smoking cessation appears as the (maximum) time elapsed since smoking cessation with never smoking as the reference. We adjusted odds ratios (with 95 % confidence intervals) for education, tooth brushing frequency, diabetes and alcohol use (g/week)

Figure 1 illustrates the effect of years since smoking cessation on the self-reported number of teeth among former-smoker men by means of adjusted (education, tooth brushing, alcohol use and diabetes) odds ratios with the number of teeth as a dichotomous outcome (‘0–27’ or ‘28–32’ teeth). As more years elapsed since smoking cessation was incorporated into the assessment of risk for fewer teeth, the estimated adjusted odds ratios steadily approached the value of one, but did not reach the level of never smokers. The corresponding analysis among women showed no association between years since smoking cessation and the number of teeth.

## Discussion

This study among middle-aged Finnish adults showed that current and former long-term smoking was associated with tooth loss. In addition, smoking cessation years seemed to lessen tooth loss, at least among men.

### Benefits of smoking cessation

Tobacco smoking is a common risk factor for numerous diseases, increasing the risk for lung cancer, cardiovascular and pulmonary diseases, as well as damaging oral health [4, 7, 11, 21]. Smoking was common in Finland in the 1960s, but has decreased drastically in recent decades due to intense smoking cessation efforts [22, 23]. This development has been especially visible among men. Today, the prevalence of smoking in Finland is amongst the lowest in Europe, a development most evident in our middle-aged study population: about one-fourth (28 %) of the participants were current smokers and one-fourth (24 %) were former smokers, whereas nearly half (48 %) were never smokers.

Few studies have specifically examined the association between smoking cessation and tooth loss, and most of these have utilised only survey data without clinically assessed number of teeth [12, 13]. Furthermore, studies are often limited to male study populations only [14, 15, 24]. Our study, in contrast, incorporated clinically assessed number of teeth with corresponding self-reported value for both men and women.

In a German population, smoking associated with a higher incidence of self-reported tooth loss among men and younger participants than among women and older participants [12]. Similarly, our findings showed a stronger association between smoking and tooth loss among men than among women. Moreover, our previous study (with clinically assessed outcome) showed a significant association between smoking and tooth loss among men only [8]. Arora et al. [13] investigated different measures of smoking history and self-reported edentulism in a large Australian cohort aged 45 and older; they found that current and former smoking associated with edentulism, but the association was weaker among former smokers than among current smokers. In line with those findings, our study among middle-aged Finns with lifelong access to subsidised dental care, revealed a slightly stronger association with tooth loss among current smokers than among former smokers.

### Time elapsed since smoking cessation

Dietrich et al. [12] found that 10 to 20 years of smoking cessation reduced the risk for tooth loss nearly to the risk level of never smokers, depending on age and gender. Similarly, Arora et al. [13] reported a decline in the risk for tooth loss with time elapsed since smoking cessation, though they also suggested that the effects of smoking may last up to 30 or more years. Our main findings, based on both self-reported and clinically assessed outcomes, regarding former smokers agree with the results of those studies: former smokers seem to be at slightly lower risk for tooth loss than current smokers, and time elapsed since smoking cessation associates positively with the number of remaining teeth among men.

We found no benefit for time elapsed since smoking cessation against tooth loss among the women, probably because of the discrepancy in smoking history between former male and female smokers. The female smokers generally smoked for fewer years, and thus had less overall exposure, than the male smokers, which causes uncertainty over the smoking cessation analyses of the female smokers. Incidentally, one must remember that smoking is uncommon among pregnant Finnish women, as many aim to avoid exposing their young children to second-hand smoke [25]. Similarly, the mean number of tobacco product units smoked per day was lower among female than among male current smokers (11.1 and 14.7, respectively). Such a discrepancy between men and women is culture dependent and was even more prominent in a study from Japan, which had to exclude women from the analyses due to exceedingly low numbers of current and former smokers [24]. Nevertheless, the findings regarding smoking cessation and clinically assessed tooth loss among 40- to 75-year-old men were similar to ours.

Krall et al. [15] properly assessed the effect of smoking cessation years on clinically assessed tooth loss in a prospective study of oral health and aging among US males. They predicted that the risk for tooth loss among former smokers would return to the level of never smokers after at least 9 to 12 years of abstinence from smoking. Similarly, we found that 10–11 years of abstinence from smoking among men could result in a greater decrease in their risk for tooth loss than could a shorter duration of abstinence from smoking.

### Strengths and weaknesses

Our comprehensive study population represents the middle-aged Finnish population well [8], and the collection of diverse and detailed information on the health of the participants allowed us to take into account several different confounders; both of these were major strengths of this study.

In addition, unlike previous studies on smoking cessation and tooth loss, we carried out more thorough analyses by utilising both self-reported and clinically assessed number of teeth to study associations among men and women. By using self-reported number of teeth (together with the clinical assessment), we attained greater fidelity with a higher number of participants in our analyses [8]. However, the assessment of self-reported number of teeth did not distinguish wisdom teeth from other teeth, which is why we decided to use a dichotomous outcome (‘0–27’ or ‘28–32’ remaining teeth) rather than a count outcome [8]. We found that if a person had fewer than 28 teeth in his or her mouth, he or she would be missing at least one tooth other than a wisdom tooth, thus implying a disease-attributable condition. Even with this high cut-off value among male and female former smokers, 57 % and 60 %, respectively, had at least 28 teeth in their mouth (based on self-reported data).

Self-reported number of teeth often serves as an easily obtainable alternative to corresponding clinical assessment. In our study, we found that overall agreement (according to Cohen’s kappa) between self-reported and clinical measurements was substantial (0.78) [26]. In addition, a total of 82 % of the participants' self-reported values were within a one-tooth margin of error from the clinically assessed value. Moreover, our parallel analyses with self-reported and clinical outcomes, with different size datasets, yielded equivalent results even though analyses with the clinical outcome were more prone to random error than were the analyses with the self-reported outcome.

One limitation of this study was its cross-sectional design with follow-up data on 46-year-olds, which rendered impossible any examination of a causal association between smoking and tooth loss. Moreover, we were unable to assess various age groups, thus limiting the generalisation of our findings to younger or older age groups.

## Conclusions

The findings of this study show that smoking has long-term effects on tooth loss even after cessation. Multifaceted tobacco-control programmes are still needed in order to reduce the burden of smoking on general and oral health.

## Abbreviations

CI: Confidence interval
NFBC1966: the Northern Finland Birth Cohort 1966
OR: Odds ratio
SD: Standard deviation
US: United States
